# Impact of allogeneic stem cell transplantation comorbidity indexes after haplotransplant using post‐transplant cyclophosphamide

**DOI:** 10.1002/cam4.4262

**Published:** 2021-09-21

**Authors:** Maxime Jullien, Corentin Orvain, Ana Berceanu, Marie‐Anne Couturier, Thierry Guillaume, Pierre Peterlin, Alice Garnier, Amandine Le Bourgeois, Marion Klemencie, Aline Schmidt, Mathilde Hunault, Etienne Daguindau, Xavier Roussel, Pascal Delepine, Gaelle Guillerm, Aurelien Giltat, Sylvie François, Sylvain Thepot, Steven Le Gouill, Marie‐C Béné, Patrice Chevallier

**Affiliations:** ^1^ Hematology Department Nantes University Hospital Nantes France; ^2^ Hematology Department Angers University Hospital Angers France; ^3^ Hematology Department Besançon University Hospital Besançon France; ^4^ Hematology Department Brest University Hospital Brest France; ^5^ Cell therapy Unit Etablissement Français du Sang – Bretagne, Site of Brest Brest France; ^6^ INSERM UMR1232 CRCINA IRS‐UN University of Nantes Nantes France; ^7^ Hematology Biology Nantes University Hospital Nantes France

**Keywords:** allogeneic hematopoietic stem cell transplantation, augmented comorbidity/age index, comorbidity/age index, haploidentical, HSCT‐CI, PTCY

## Abstract

**Background:**

Three different scoring systems have been developed to assess pre‐transplant comorbidity in allogeneic hematopoietic stem cell transplantation (Allo‐HSCT): the Hematopoietic Cell Transplantation‐Specific Comorbidity Index, the Comorbidity/Age index, and the Augmented Comorbidity/Age index. All were devised to predict overall survival (OS) and disease‐free survival (DFS) survivals and non‐relapse mortality (NRM) in patients receiving HLA‐matched Allo‐HSCT, but their performance has scarcely been studied in the haploidentical Allo‐HSCT setting with post‐transplant cyclophosphamide, a procedure in constant expansion worldwide.

**Methods:**

To address this issue, their impact on survivals and NRM was examined in a cohort of 223 patients treated with haploidentical Allo‐HSCT in four different centers.

**Results:**

With a median follow‐up of 35.6 months, 3‐year OS, DFS, and NRM were 48.1% ± 4%, 46.3% ± 4%, and 30.0% ± 3%, respectively. No impact was found for any of the three comorbidity scores in univariate analysis. In multivariate analyses, the only three factors associated with lower OS were DRI (*p* < 0.001), an older age of recipients (≥55 years old, *p* = 0.02) and of donors (≥40 years old, *p* = 0.005). Older donor age was also associated with lower DFS and higher NRM.

**Conclusion:**

The comorbidity scores do not predict survivals nor NRM in haploidentical Allo‐HSCT with PTCY, suggesting that pre‐transplant comorbidities should not be a contra‐indication to this procedure.

## Graphical abstract

Three different scoring systems have been developed to assess pre‐transplant comorbidity in allogeneic hematopoietic stem cell transplantation: the Hematopoietic Cell Transplantation‐Specific Comorbidity Index, the Comorbidity/Age index, and the Augmented Comorbidity/Age index, but their performance has scarcely been studied in the haploidentical stem cell transplantation setting with post‐transplant cyclophosphamide, a procedure in constant expansion worldwide. To address this issue, their impact was examined in a cohort of 223 patients from four French centers treated with haploidentical stem cell transplantation. No impact was found for any of the three comorbidity scores in this study, suggesting that pre‐transplant comorbidities should not be a contra‐indication to this procedure.

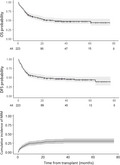

## 1 INTRODUCTION

Allogeneic hematopoietic stem cell transplantation (Allo‐HSCT) is a curative therapeutic modality for a great number of hematological disorders. Despite improvements over the last decade, this procedure is still associated with a high non‐relapse mortality rate (NRM).^1^ The development of tools to enable a better selection of patients liable to benefit the most from Allo‐HSCT is, therefore, necessary. In this regard, three different scoring systems have been developed to assess pre‐transplant comorbidities. The Hematopoietic Cell Transplantation‐Specific Comorbidity Index (HCT‐CI) was developed in 2005 by Sorror et al.,^2^ from the retrospective analysis of a cohort of 1055 patients having received non‐ablative or ablative conditionings followed by hematopoietic cell grafts from related or unrelated donors. With a threshold of 3, the HCT‐CI allowed to predict NRM and survival from a 17‐item scoring system, with weights assigned to each comorbidity according to its prognostic significance in a Cox proportional hazard model. The prognostic value of HCT‐CI was augmented by the addition of the age of the recipient (< or ≥40 years old), establishing the Comorbidity/Age index (C/AI) (< vs. ≥3), based on a large multicenter retrospective study including patients treated with Allo‐HSCT after any type of conditioning regimen and having received grafts from HLA‐matched related or unrelated donors.^3^ More recently, this score has been further improved with the addition of pre‐transplant ferritin (< or ≥2500 µg/L) and albumin (<3 g/dl vs. 3–3.5 g/dl vs. ≥3.5 g/dl) serum levels, as well as platelet counts (< vs. ≥100 × 10^9^/L), constituting the Augmented Comorbidity/Age index (AC/AI) that uses a threshold of 4.^4^ The latter was also based on a large retrospective study incorporating HLA‐matched related or unrelated recipients.

Haploidentical Allo‐HSCT (Haplo‐SCT) using post‐transplant cyclophosphamide (PTCY) is a procedure in constant expansion worldwide.^5^ The HCT‐CI has been already explored in this setting yet with conflicting results as it has been shown to impact^6^, ^7^, ^8^, ^9^ or not^10^, ^11^, ^12^, ^13^, ^14^ survivals and/or NRM. Interestingly, the HCT‐CI was found to influence NRM without impacting survivals^7^ or conversely influence survivals without impacting NRM.^8^ It has also been identified to predict graft‐versus‐host disease (GVHD)^13^ or relapse.^15^

Conversely, the performance of C/AI and AC/AI scores has been poorly evaluated after Haplo‐SCT. To our knowledge, only Elsawy et al.^16^ have demonstrated so far a non‐statistically significant impact on NRM and OS of the AC/AI score in a cohort of 117 patients receiving Haplo‐SCT, potentially because of an insufficient statistical power.

We report here the results of a multicenter retrospective study designed to assess and compare the value of the three comorbidity scores (HCT‐CI, A/CI, and CA/CI) in terms of predicting survivals and NRM in 223 patients having received Haplo‐SCT with PTCY.

## 2 PATIENTS AND METHOD

### 2.1 Patients

This study has included all consecutive recipients of a Haplo‐SCT with PTCY in four different centers between October 2013 and January 2020. The main objective was to assess and compare the influence of the three comorbidity scores currently used in allotransplant on survivals and NRM. HCT‐CI and A/CI were calculated as previously described from each patient’s medical file.^2^, ^3^, ^17^ All data were reviewed for each patient in each center by a trained operator blinded to the patient’s outcome. All data were available to provide accurate HCT‐CI scores. Pre‐transplant ferritin and albumin levels as well as platelet counts were evaluated at the time of pre‐transplant check‐up or just before conditioning (median from transplant: 20 days, range: 4–49), allowing to calculate the AC/AI^4^ for all cases. The secondary objective was to decipher which factors can predict outcomes after transplant by comparing comorbidity scores with other parameters related to each patient or donor. Various conditionings were used, either myeloablative, sequential, or reduced‐intensity, including Baltimore‐based regimen with fludarabine and clofarabine,^18^, ^19^ clofarabine/fludarabine regimen,^20^ or TBF regimen with one or two days of thiotepa.^21^ However, all patients received PTCY, cyclosporine, and mycophenolate mofetil as graft‐versus‐host disease (GVHD) prophylaxis. All patients had provided informed consent for data entry into the European Bone Marrow Transplantation group registry database for observational/retrospective studies. The study was performed in accordance with the Declaration of Helsinki. The institutional review boards of the four centers reviewed and approved this study.

### 2.2 Statistical analyses

The clinical and biological outcomes studied were overall survival (OS), disease‐free survival (DFS), and NRM. OS was defined as the time from day 0 of Haplo‐SCT to death or last follow‐up. DFS was defined as time from day 0 of Haplo‐SCT to time without evidence of relapse or disease progression censored at the date of death or last follow‐up. NRM was defined as death from any cause without previous relapse nor progression. Probabilities of OS and DFS were calculated using the log‐rank test and Kaplan‐Meier graphical representation. The cumulative incidence of NRM was calculated using relapse or progression as competing risks.

Univariate analyses were performed using the log‐rank test. Characteristics considered for univariate analysis were as follows: gender (male vs. female), ethnicity (Caucasians vs. others), recipient age (< or ≥median (=55) years old (yo), and <or ≥40 yo), disease (lymphoid vs. myeloid), disease‐risk index (DRI)^22^ (low/intermediate vs. high/very high), donor age (< or ≥median (=40) yo), pre‐transplant ferritin (< or ≥2500 µg/L) and albumin (<3 g/dl vs. 3–3.5 g/dl vs. >3.5 g/dl) serum levels, pre‐transplant platelet counts (< vs. ≥100 10^9^/L), HCT‐CI (< vs. ≥3), C/AI (< vs. ≥3), and AC/AI (< vs. ≥4). Multivariate analyses were performed using the Cox proportional hazard model. Factors with a *p* value <0.20 by univariate analysis or of interest for the study were included in multivariate analysis. All tests were two‐sided and *p* values <0.05 were considered as indicating a statistically significant association. Statistical analyses were performed in July 2020 using the R software (version 4.0.2).

## 3 RESULTS

### 3.1 Patient characteristics

Patient characteristics are summarized in Table 1. There were 136 males and 87 females with a median age of 55 years, with 174 patients over 40. The majority of patients had a myeloid disease (*n* = 155) and received a reduced intensity regimen (*n* = 161, myeloablative *n* = 30; sequential *n* = 32). Respectively, 133 and 90 patients had low/intermediate and high/very high DRI. Donors had a median age of 40.8 years (19–72). Median HCT‐CI, C/AI, and AC/AI scores were 2 (0–8), 3 (0–9), and 3 (0–11), respectively. The HCT‐CI score was <3 in 139 patients, CA/I was <3 in 113 patients, while the AC/AI score was <4 in 112 cases. Of note, the impact of ECOG performance status could not be evaluated in this series as only four patients had a score >1.

**TABLE 1:** Patient characteristics

*N* = 223	
Centers	
#1/ #2 / #3 / #4	127/45/29/22
Gender: male/female	136/87
Ethnic group: Caucasian/other	203/20
Median age at transplant: years (range)	55 (16–71)
Age ≥40 year old	174
Median follow‐up for alive patients: months (range)	35.6 (6–77)
Disease: myeloid/lymphoid	155/68
AML/MDS/MPS/BPDCN	111/27/14/3
ALL/NHL/HL/CLL	18/25/15/10
Disease‐risk index: low/intermediate/high/very high	14/119/70/20
HCT‐CI: median (range)	2 (0–8)
HCT‐CI <3 / ≥3	139/84
Comorbidity/Age Index (C/AI)	Median 3 (0–9)
C/AI ≤2/3–4/≥5	110/83/30
Pre‐transplant parameters	
Serum ferritin (SF): median (range), µg/L	1554 (13–11,624)
SF <1500	110
SF 1500–2500	49
SF>=2500	65
Platelets (Plts): median (range), ×10^9^/L	116 (5–895)
Plts <100 × 10^9^/L	93
Albumin (Alb): median (range), g/L	39 (18–52)
Alb <3	15
Alb 3–3.5	36
Alb >=3.5	173
Augmented Comorbidity/Age Index: median (range) AC/AI	3 (0–11)
AC/AI <4/≥4	113/111
Conditioning: MAC	30
T2BF	28
Fluda/TBI 12 Grays	2
RIC	161
Baltimore with fludarabine	65
Baltimore with clofarabine	32
CloB2A1	29
T1BF	35
Sequential	32
GVHD prophylaxis: PTCY/cyclosporine/MMF	223
Haplodonor	
Median age: years (range)	40.8 (19.4–71.7)
≥40 year old	106
Son/daughter	100 (67/33)
Brother/sister	90 (50/40)
Father/mother	23 (16/7)
Nephew/niece	6 (5/1)
Female to male	47
Source of graft	
Peripheral blood stem cells	207
Bone marrow	16
CD34+ stem cells infused: median (range) 10^6^/kg of recipient (*n* = 160)	6.73 (2.88–19.94)
CD3+ cells infused: median (range) 10^7^/kg of recipient (*n* = 156)	23.95 (3.93–66.75)
CD45+ cells infused: median (range) 10^8^/kg of recipient (*n* = 129)	8.08 (3.2–25.36)

Abbreviations: ALL, acute lymphoblastic leukemia; AML, acute myeloid leukemia, MDS, myelodysplastic syndrome; BPDCN, blastic plasmacytoid dendritic cell neoplasm; CLL, chronic, lymphocytic leukemia; CloB2A1, clofarabine, busulfan, antithymoglobuline; GVHD, graft‐versus‐host disease; HCT‐CI, Hematopoietic Cell Transplantation‐Specific Comorbidity Index; HL, Hodgkin lymphoma; MAC, myeloablative; MPS, myeloproliferative syndrome; NHL, non‐Hodgkin lymphoma; PTCY, post‐transplant cyclophosphamide, MMF, mycophenolate mofetil; RIC, reduced‐intensity conditioning; T1BF/T2BF, thiotepa 1 or 2 days, busulfan, fludarabine.

### 3.2 Outcomes of the whole cohort

**FIGURE 1:**
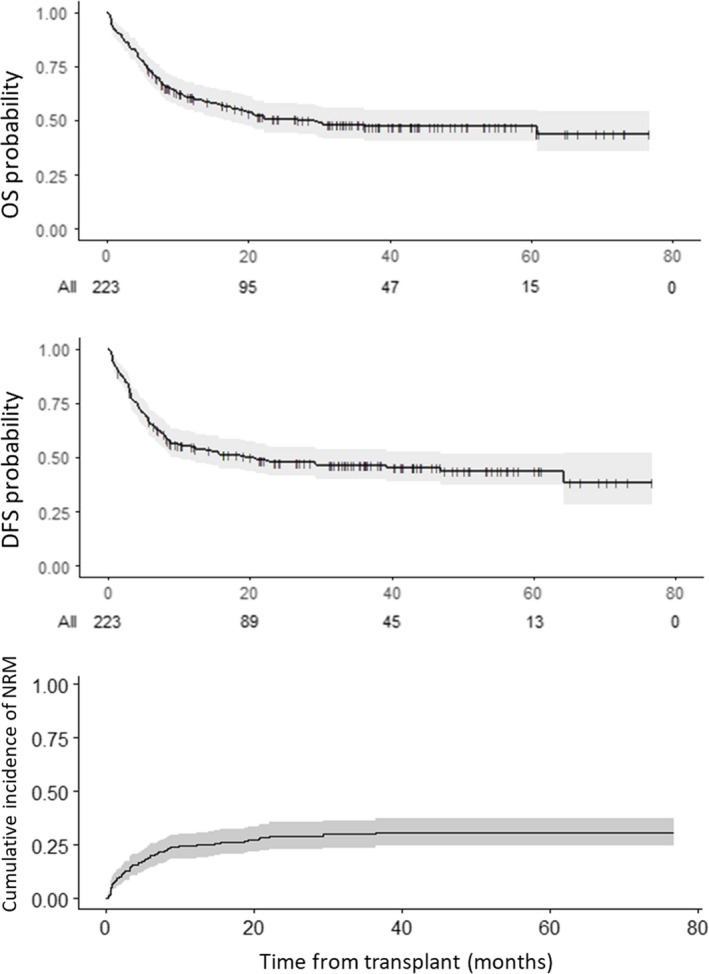
Overall survival (OS), disease‐free survival (DFS), and non‐relapse mortality (NRM) of the whole cohort

With a median follow‐up for alive patients of 35.6 months (range 6–77), 3‐year OS, DFS, and NRM were 48.1% ± 4%, 46.3 ± 4%, and 30.0% ± 3%, respectively (Figure 1). At the time of analysis, 110 deaths had occurred, due to relapse (*n* = 45), sepsis (*n* = 36), GVHD (*n* = 13), or organ toxicity (*n* = 11). The cause of death remained unknown for five patients.

### 3.3 Univariate analysis

Better 3‐year OS and DFS were associated with a low/intermediate DRI (57.7% ± 5% vs. 31.5% ± 6% *p* < 0.001, and 54.6% ± 5% vs. 32.0% ± 6% *p* < 0.001, respectively), a donor under 40 yo (58.7% ± 5% vs. 36.2% ± 5% *p* = 0.004, and 56.0% ± 5% vs. 35.7% ± 5% *p* = 0.01, respectively) and a higher albumin level (>3 g/dl) at time of transplant (<3g/dl: 30.0% ± 13% vs. 3–3.5: 44.6% ± 9% vs. >3.5: 50.5% ± 4%, *p* = 0.02, and 33.3% ± 12% vs. 46.2% ± 8% vs. 47.8% ± 4%, *p* = 0.02). Recipients younger than 55 or treated for a lymphoid disease had better OS (54.4% ± 5% vs. 42.1% ± 5%, *p* = 0.04, and 58.4% ± 6% vs. 43.4% ± 4%, *p* = 0.05, respectively) but not better DFS. Finally, a lower NRM was observed in younger recipients (20.7% ± 4% vs. 39.2% ± 5%, *p* = 0.005) and patients transplanted with a younger donor (18.5% ± 4% vs. 42.6% ± 5%, *p* < 0.001). The main cause of higher NRM in older recipients or patients with older donors was sepsis (55% and 53.6%, respectively; Table 2).

**TABLE 2:** Univariate analysis

Variable	OS			DFS			NRM		
	HR	95% CI	*p*	HR	95% CI	*p*	HR	95% CI	*p*
DRI									
High/very high versus low/intermediate	2.12	1.42–3.15	**<0.001**	2.05	1.39–3.01	**<0.001**	1.10	0.66–1.82	0.73
Disease									
Myeloid versus lymphoid	1.64	1.05–2.57	0.03*	1.52	0.99–2.33	0.05	1.63	0.90–2.97	0.11
Recipient gender									
Male versus female	1.12	0.75–1.64	0.58	0.98	0.68–1.42	0.92	1.28	0.77–2.15	0.34
Recipient ethnic group									
Caucasian versus other	1.63	0.76–3.52	0.21	1.29	0.65–2.54	0.47	1.21	0.48–3.10	0.68
Recipient age									
≥55 yo versus <55 yo	1.49	1.02–2.17	0.04*	1.38	0.96–1.99	0.08	2.09	1.25–3.49	0.005**
Donor age									
≥40 yo versus <40 yo	1.31	0.82–2.09	0.25	1.18	0.76–1.84	0.46	2.02	0.98–4.15	0.06
≥40 yo versus <40 yo	1.73	1.19–2.53	0.004**	1.58	1.10–2.28	0.01*	2.41	1.45–4.03	**<0.001**
Albumin*									
<3 versus >3.5	2.28	1.18–4.41	0.02*	2.24	1.17–4.33	0.02*	1.35	0.85–2.12	0.2
3–3.5 versus >3.5	1.29	0.78–2.12	0.32	1.15	0.70–1.89	0.57	1.28	0.67–2.44	0.45
Ferritin**									
≥2500 versus <2500	1.12	0.74–1.68	0.6	1.11	0.75–1.65	0.59	0.88	0.51–1.53	0.66
HCT‐CI									
≥3 versus <3	1.35	0.93–1.97	0.12	1.30	0.90–1.87	0.16	1.25	0.77–2.03	0.37
C/AI									
≥3 versus <3	1.16	0.79–1.68	0.45	1.07	0.74–1.53	0.73	1.05	0.65–1.7	0.85
AC/AI									
≥4 versus <4	1.35	0.93–1.97	0.12	1.24	0.86–1.78	0.25	1.26	0.77–2.05	0.36

Bold indicates significant values.

Abbreviations: AC/AI, Augmented Comorbidity/Age index; C/AI, Comorbidity/Age index; DFS, disease‐free survival; DRI, disease‐risk index. Yo, years old; HCT‐CI, Hematopoietic Cell Transplantation‐Specific Comorbidity Index; NRM, non‐relapse mortality; OS, overall survival.

*g/dl; **µg/L.

Donor age was the sole factor predicting both survivals and NRM. OS, DFS, and NRM were not impacted by gender, ethnicity, pre‐transplant ferritin level, platelet counts, nor any of the three comorbidity scores. No center effect was observed in this series.

### 3.4 Multivariate analysis

High/very high DRI remained associated with lower OS (HR: 2.20, 95%CI: 1.41–3.43, *p* < 0.001) and DFS (HR: 2.09, 95%CI: 1.41–3.10, *p* < 0.001). An older age of recipients remained associated with lower OS (HR: 1.67, 95%CI: 1.10–2.52, *p* = 0.02) and higher NRM (HR: 2.18, 95%CI: 1.30–3.66, *p* = 0.003). Again, older donor remained the sole factor associated with both lower survivals (OS, HR: 1.81, 95%CI: 1.20–2.74, *p* = 0.005, and DFS, HR: 1.55, 95%CI: 1.04–2.30, *p* = 0.03) and higher NRM (HR: 2.50, 95%CI: 1.50–4.18, *p* < 0.001) Table 3.

**TABLE 3:** Multivariate analysis

Multivariate analysis		OS			DFS			NRM		
Variable		HR	95% CI	*p*	HR	95% CI	*p*	HR	95% CI	*p*
DRI	High/very high versus Low/intermediate	2.20	1.41–3.43	**<0.001**	2.09	1.41–3.10	**<0.001**	—	—	—
Disease	Myeloid versus lymphoid	1.26	0.77–2.08	0.36	—	—	—	—	—	—
Recipient age	≥55 yo versus <55 yo	1.67	1.10–2.52	**0.02**	—	—	—	2.18	1.30–3.66	**0.003**
Donor age	≥40 yo versus <40 yo	1.81	1.20–2.74	**0.005**	1.55	1.04–2.30	**0.03**	2.50	1.50–4.18	**<0.001**
Albumin	<3 versus >3.5 g/dl	1.19	0.57–2.47	0.65	1.31	0.63–2.73	0.46	—	—	—

Bold indicates significant values.

Abbreviations: DFS, disease‐free survival; DRI, disease‐risk index; NRM, non‐relapse mortality; OS, overall survival; yo, years old.

## 4 DISCUSSION

The large multicenter retrospective study reported here was designed to assess and compare the validity of three available pre‐transplant comorbidity scores for predicting survivals and NRM after Allo‐HSCT in the particular setting of Haplo‐SCT with PTCY. This retrospective analysis included 223 consecutive cases and suggests that none of the three comorbidity scores (HCT‐CI, C/AI, and AC/AI) was able to predict survivals or NRM in this population. Considering HCT‐CI, our results are consistent with previous published studies.^10^, ^11^, ^12^, ^13^, ^14^ It is also the case considering C/AI and AC/AI, although only one study, to the best of our knowledge, has explored their value after Haplo‐SCT. Indeed, Elsawy et al.^16^ reported no statistically significant differences in terms of OS nor NRM for patients with high AC/AI in a cohort of 117 patients receiving Haplo‐SCT (respectively HR: 1,19, *p* = 0.60 and HR: 1.66, *p* = 0.08 for AC/AI ≥4 vs. 0–3). The discrepancy with other reports showing an impact of HCT‐CI^6^, ^7^, ^8^, ^9^ may likely be due to the retrospective nature and potential selection biases for all of these studies including ours. However, our cohort is currently the largest one having evaluated the impact of C/AI and AC/AI in Haplo‐SCT. One of the weakness of the study by Elsawy et al. was clearly too small a cohort, as it has been reported that at least 200 patients are required to validate the pertinence of the AC/AI score.^23^ The fact that the prognostic value of HCT‐CI is not improved by the two other scores strengthens the absence of impact of pre‐transplant comorbidities compared to other studies.^6^, ^7^, ^8^, ^9^ The question is why none of the scores is able to predict outcomes in Haplo‐SCT followed by PTCY. As such, an evaluation of individual comorbidities would be of interest to possibly identify those specifically associated with NRM in the particular setting of Haplo‐SCT. Indeed, in the matched setting, it was shown that comorbidities may exert effects on NRM but unique to particular conditioning regimens, suggesting that regimen selection should be driven, in part, by specific comorbidities.^24^ Unfortunately, details regarding all individual comorbidities were not available in the present study, which is one of its limitations.

It seems that HCT‐CI, C/AI, and AC/AI inadequately capture pre‐transplant comorbidities in older adults.^13^ In this population, factors, such as poor social support, cognitive limitation, slow walk‐speed, poor basic activities of daily living, and poor mental health, have been reported to be associated with adverse outcomes after Allo‐HSCT,^25^, ^26^, ^27^, ^28^ and the application of comprehensive geriatric assessments allows for the detection of vulnerabilities not captured by comorbidity scores.^29^, ^30^, ^31^, ^32^ Finally, socio‐economic conditions seem to play an important role in transplant‐associated mortality. For example, a poor access to care in addition to individual comorbidities was associated with NRM in a recent study.^33^

It seems also that a better stratification could be obtained by combining HCT‐CI with the EBMT score modified model.^34^, ^35^ Finally, if the biomarker IL‐6 may be a surrogate marker for HCT‐CI,^36^ it has been shown to predict outcomes contradictorily to this comorbidity score in Haplo‐SCT.^37^ Thus, our results suggest that other factors should be taken into account and that pre‐transplant comorbidity scores are not sufficient themselves to contra‐indicate the Haplo‐SCT procedure. More importantly, comorbidities calculated on the basis of these three scores should not represent exclusion criteria when it comes to propose protocol participation in the context of Haplo‐SCT. Thus, an appropriate evaluation of a larger sample of patients in a prospective fashion and including other variables, especially geriatric assessments, is warranted.

Interestingly, we could evaluate the value of such parameters as pre‐transplant ferritin and albumin levels. None of these two factors were able to predict survivals nor NRM in multivariate analyses. In fact, if data exist for matched transplantation showing some influence for both markers,^4^, ^38^ there are no available results for Haplo‐SCT. Thus, other studies definitely need to be conducted to evaluate precisely the role of these markers in this particular setting. It should be interesting also to better assess the role of post‐transplant ferritin levels and/or of iron chelation in this context, as it has been done for matched transplants.^39^

One of the most spectacular results of this large cohort is that donor age turned out to be the only adjustable factor predicting both survivals and NRM, suggesting its crucial importance in the setting of Haplo‐SCT, especially when it comes to donor choice. The impact of donor age has been evaluated in the context of HLA‐matched transplantation with conflicting results, as it has been found to influence^40^, ^41^ or not^42^ outcomes depending mainly on the cut‐off chosen. Similar conflicting data have been observed after Haplo‐SCT with PTCY.^8^

In conclusion, it seems that the HCT‐CI, C/AI, and AC/AI scores do not predict survivals nor NRM in Haplo‐SCT with PTCY, suggesting that pre‐transplant comorbidities should not be a contra‐indication to this procedure. As donor age is the only factor predicting survivals and NRM in this series, the selection of a younger donor should be the preferred choice whenever possible for these patients. Further analyses are needed to validate these results.

## CONFLICT OF INTERESTS

The authors declare no conflict of interest.

## ETHICAL STANDARDS STATEMENT

All procedures followed were in accordance with the ethical standards of the responsible committee on human experimentation (institutional and national) and with the Helsinki Declaration of 1975, as revised in 2008.

## STATEMENT OF INFORMED CONSENT

Informed consent was obtained from all patients for being included in the study.

## Data Availability

The data that support the findings of this study are available from the corresponding author, PC, upon reasonable request.
